# Differential Expression and Clinical Significance of Transforming Growth Factor-Beta Isoforms in GBM Tumors

**DOI:** 10.3390/ijms19041113

**Published:** 2018-04-08

**Authors:** Laurent-Olivier Roy, Marie-Belle Poirier, David Fortin

**Affiliations:** 1 Department of Pharmacology, Université de Sherbrooke, Sherbrooke, QC J1H 5N4, Canada; Laurent-Olivier.Roy@USherbrooke.ca; 2 Department of Surgery, Université de Sherbrooke, Sherbrooke, QC J1H 5N4, Canada; Marie-Belle.Poirier@USherbrooke.ca

**Keywords:** glioblastoma, transforming growth factor-beta, overall survival, post-reoperation survival, progression-free survival

## Abstract

Glioblastoma (GBM) represents the most common and aggressive malignant primary brain tumors in adults. Response to standard treatment is transitory and the survival of clinical trial cohorts are little more than 14 months. GBM are characterized by excessive proliferation, invasiveness, and radio-/chemoresistance features; which are strongly upregulated by transforming growth factor-beta (TGF-β). We hypothesized that TGF-β gene expression could correlate with overall survival (OS) and serve as a prognostic biomarker. TGF-β_1_ and -β_2_ expression were analyzed by qPCR in 159 GBM tumor specimens. Kaplan–Meier and multivariate analyses were used to correlate expression with OS and progression-free survival (PFS). In GBM, TGF-β_1_ and -β_2_ levels were 33- and 11-fold higher respectively than in non-tumoral samples. Kaplan–Meier and multivariate analyses revealed that high to moderate expressions of TGF-β_1_ significantly conferred a strikingly poorer OS and PFS in newly diagnosed patients. Interestingly, at relapse, neither isoforms had meaningful impact on clinical evolution. We demonstrate that TGF-β_1_ is the dominant isoform in newly diagnosed GBM rather than the previously acknowledged TGF-β_2_. We believe our study is the first to unveil a significant relationship between TGF-β_1_ expression and OS or PFS in newly diagnosed GBM. TGF-β_1_ could serve as a prognostic biomarker or target affecting treatment planning and patient follow-up.

## 1. Introduction

Malignant gliomas are the most common type of primary central nervous system neoplasms and represent 80% of all malignant brain tumors [1]. According to the 2016 World Health Organization (WHO) classification, they are classified as either isocitrate dehydrogenase 1/2 (IDH-1/2) wild-type or mutated tumors and classically encompass an astrocytic or oligodendroglial histological subtype. Their final stratification is based on the molecular diagnosis integrating IDH-1 status as well as other markers (alpha thalassemia/mental retardation syndrome X-linked (ATRX), 1p-19q co-deletion, p-53) and histological characteristics [2]. Glioblastoma (GBM) accounts for 54% of all gliomas and has the most aggressive phenotype, even though it is a very heterogeneous class of tumor in terms of clinical behavior. In a clinical trial published by Stupp and colleagues, survival of newly diagnosed GBM was 14.6 months when treated with standard therapy consisting of tumor resection followed by radiotherapy with concomitant and adjuvant temozolomide (Stupp protocol) [3]. However, because of the exceedingly infiltrative behavior of neoplastic glial cells in the brain parenchyma, complete resection is unattainable and the associated radio- and chemoresistant features of GBM usually lead to a transitory response; thus, tumor recurrence is inevitable [4,5]. Despite decades of research, the first-line therapy established by Stupp and colleagues remains the gold standard whereas consensus regarding second-line therapy has yet to be reached. Heterogeneity in treatment response between patients makes it very difficult to pinpoint an effective therapeutic strategy at relapse. Indeed, molecular refinements have made it clear that this disease is extremely heterogeneous, with different molecular and prognostic subtypes.

In order to better understand the divergence between patients and uncover potential therapeutic strategies, the neuro-oncology community has begun researching for molecular biomarkers and targets that could help predict the aggressiveness of GBM tumors as well as benefit treatment [6,7]. Endeavors in genomics such as The Cancer Genome Atlas (TCGA) or in molecular biology have shed light on new subgroups of patients and novel molecular targets. Indeed, identification of GBM subclasses is now considered in therapeutic strategies [8]. Likewise, several targets have received great interest such as tyrosine kinases, integrins, vascular endothelial growth factor (VEGF), and mitogen-activated protein kinases (MAPKs) [9,10,11].

Another appreciated target is the transforming growth factor-beta (TGF-β), a cytokine that has five different isoforms, three of which are expressed in humans. TGF-β signals through two serine/threonine kinase receptors to activate messenger proteins (SMADs) and induce the expression of several genes associated with a myriad of functions in the establishment and progression of GBM [12]. Indeed, TGF-β signaling is associated with proliferation, renewal of stem-like cell population and invasion [13,14]. Through interaction with VEGF and fibroblast growth factor (FGF) signaling, TGF-β also contributes to angiogenesis to harmonize blood supply with the extremely fast GBM growth [15,16]. Moreover TGF-β can promote radioresistance and concealment from the immune surveillance which contributes to poor clinical response and tumor relapse [17,18]. Furthermore, prior studies by the groups of Yamada, Kjellman, and Bayin showed that TGF-β protein and both receptors are abundantly expressed in malignant glial tumors and that TGF-β_1_ and -β_2_ mRNA expression correlated with tumor grade [19,20,21]. Rodòn and collaborators also demonstrated that TGF-β was regulated in an autocrine fashion through cAMP-responsive element-binding protein 1 (CREB1) in GBM [22]. Finally, work by Bruna and colleagues provided evidence that high TGF-β signaling activity was associated with poor prognosis [23].

Considering the multiple oncogenic features of TGF-β, we hypothesized that its expression levels might inversely correlate with overall survival (OS) or other clinical surrogates, and could also eventually serve as a therapeutic target. A study by Frei and collaborators has recently assessed the association between TGF-β and survival. However, perhaps according to a smaller cohort or a different methodological analyses, they did not unveil significant correlations [24]. For this work, we initially queried the TCGA dataset to investigate this potential relation in GBM. We then measured the expression levels of TGF-β isoforms mRNA in human malignant glioma specimens kept in our biobank. The results were then correlated with clinical data to seek a relation between expression levels and clinical surrogate.

## 2. Results

### 2.1. TGF-β Expression in the TCGA Cohort

**Figure 1:**
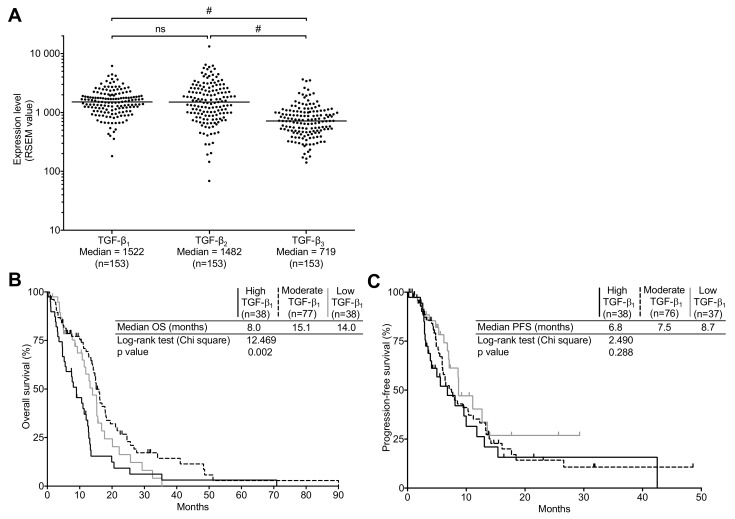
Transforming growth factor-beta (TGF-β) expression and correlation of TGF-β_1_ mRNA levels with survival in the the Cancer Genome Atlas (TCGA) cohort. (**A**) Comparison of mRNA levels (RSEM values) of all three TGF-β isoforms in newly diagnosed glioblastoma (GBM). The black line marks the median in each group. Values are represented on a logarithmic scale. #, *p* < 0.0001; ns, not significant. Kaplan–Meier estimates of (**B**) overall survival, and (**C**) progression-free survival according to three subgroups of TGF-β_1_ expression (high, black line; Moderate, dashed line; low, gray line). The progression-free survival data was unavailable for two patients.

As a first step to this study, we used the publicly available TCGA datasets to analyze the expression of TGF-β isoforms in relation to OS and PFS in GBM tumors. Hence, we used the data of 168 tumor specimens. We stratified this cohort as either newly diagnosed or recurrent GBM. This yielded two subgroups of 153 (median age: 60 years) and 13 patients (median age: 56 years), respectively. Because of the very low number of recurrent tumors and given the high inter-patient variability in TGF-β expressions as well as other clinical surrogates, further analyses were omitted for this group. In newly diagnosed tumors, the expression levels of TGF-β isoforms were found to be greatly heterogenous. Whereas there was no meaningful difference between TGF-β_1_ and -β_2_ median mRNA levels, both isoforms were significantly more expressed than TGF-β_3_ (*p* < 0.0001, Figure 1A), which is congruent with previous reports [20].

Furthermore, TGF-β_1_ was significantly more expressed in the mesenchymal subtype whereas expression levels were similar between classical, neural, and proneural newly diagnosed GBM. Moreover, we found no relevant difference in TGF-β_2_ levels between classical, mesenchymal, and neural newly diagnosed GBM, but this isoform was the least expressed in the proneural subtype. Likewise, TGF-β_3_ expression did not significantly differ in the classical and mesenchymal subtype whereas the neural and proneural newly diagnosed GBM notably had lower levels (Figure S1).

**Figure 2:**
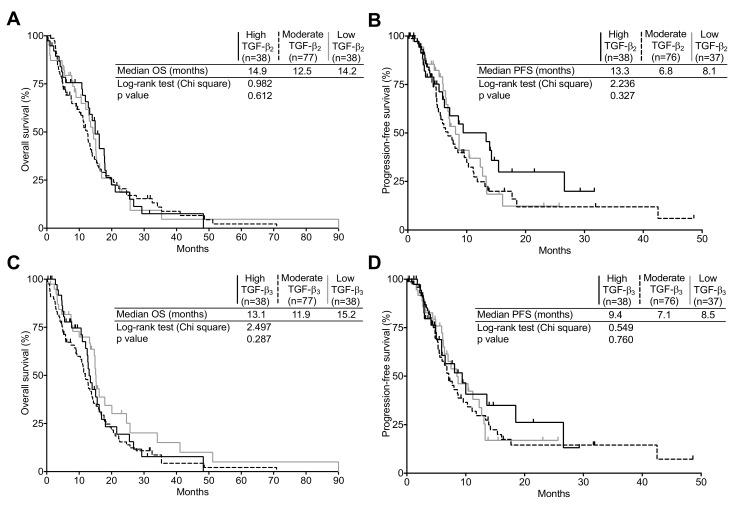
Correlation of TGF-β_1_ and -β_3_ mRNA expression with survival in the TCGA cohort. (**A**,**B**) Kaplan–Meier estimates of overall survival and progression-free survival according to three subgroups of TGF-β_2_ or (**C**,**D**) TGF-β_3_ expression (high, black line; moderate, dashed line; low, gray line). The progression-free survival data was unavailable for two patients.

To assess the correlation of TGF-β expression with OS and progression-free survival (PFS), expression values for TGF-β isoforms of both newly diagnosed and recurrent GBM were stratified in the following subgroups: high, moderate, and low, defined using the 75th and 25th percentiles as cut off values (high ≥ 75th > moderate ≥ 25th > low). As seen in Figure 1B,C, in newly diagnosed tumors, patients expressing high TGF-β_1_ levels presented a significantly poorer OS, but not PFS, than patients expressing either moderate or low TGF-β_1_ levels. However, neither TGF-β_2_ nor TGF-β_3_ expression presented a significant correlation to OS or PFS in newly diagnosed GBM (Figure 2). The median OS was 8.0 months in high TGF-β_1_ expressing GBM compared to 15.1 and 14.0 months for moderate and low TGF-β_1_ expressing tumors respectively (*p* = 0.002). We found no correlation between PFS and either TGF-β_1_ or -β_2_ expression.

Based on those results, we assessed whether TGF-β mRNA expression correlated with pathway activity. In newly diagnosed expression of TGF-β target genes PAI-1 and PDGFB significantly correlated with TGF-β_1_ levels. We found no relationship between TGF-β_2_ levels and PAI-1 or PDGFB. However, although the impact is not strong, TGF-β_3_ expression correlated with PDGFB only in newly diagnosed tumors (Figure S2).

### 2.2. Patient Characteristics

The principal characteristics of our cohort are summarized in Table S1. Out of a total of 159 GBM specimens, the newly diagnosed GBM represented a subgroup of 95 patients in which the median age was 62 years. Most of these patients were treated with the standard Stupp protocol as first-line treatment and the median OS was 13.9 months. The other subset of 64 patients were relapsing tumors, out of which seven were secondary GBMs with history of prior lower grade glioma, presenting a median age of 53 years at recurrence. While the second line therapy varied, the median OS was 24.6 months for this cohort. Thirteen paired specimen were available for analysis (from patient that underwent a second surgery). However, as many of these patients were referred to our center at relapse, after a first surgery and first-line treatment, the newly diagnosed tumor sample was unavailable for most of these patients.

We elected to analyze both subgroups (newly diagnosed and recurrent GBM) distinctively to take into account disease progression and the effect of treatments, as newly diagnosed tumors were naive to radio- and chemotherapy and gene expression might have been affected by the first-line therapy in the recurrent cohort.

### 2.3. TGF-β_1_ and -β_2_ Expression in GBM Patients Compared to Non-Tumoral Brain Samples

**Figure 3:**
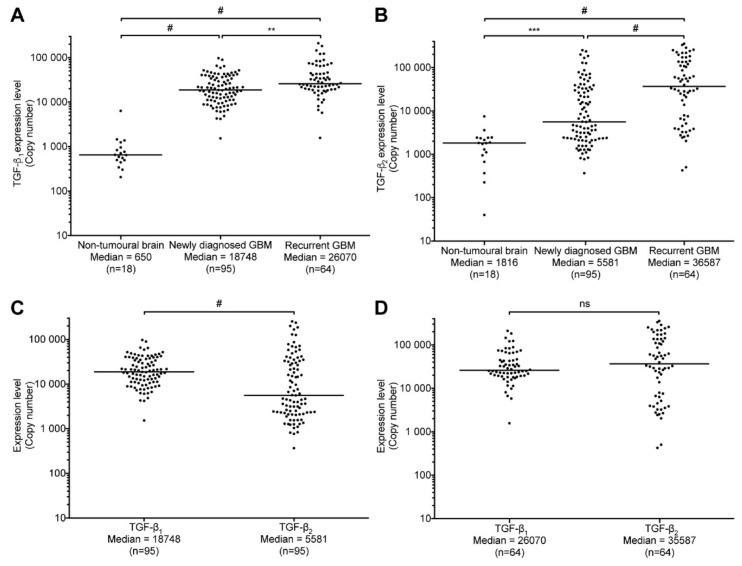
TGF-β expression in our clinical series. (**A**) Comparison of TGF-β_1_ and (**B**) TGF-β_2_ mRNA levels (copy number values) in 95 newly diagnosed and 64 recurrent GBM as well as in 18 non-tumoral brain samples. Comparison of TGF-β_1_ and -β_2_ mRNA levels (copy number values) in newly diagnosed (**C**) and recurrent GBM (**D**). The black line marks the median in each group. Values are represented on a logarithmic scale. **, *p* < 0.01; ***, *p* < 0.001; #, *p* < 0.0001; ns, not significant.

Based on the results from our analysis of TGCA data, we elected to measure TGF-β_1_ and -β_2_ mRNA levels in tumor specimens from our biobank. TGF-β_3_ was not analyzed since it was the least expressed isoform and did not correlate with OS; this decision was made in order to maximize the availability of our samples for the analysis of other genes. TGF-β_1_ and -β_2_ mRNA levels were measured using quantitative real-time PCR in 159 tumor specimens as well as in 18 non-tumoral brain tissues. As was observed within the TGCA samples, the expression of TGF-β_1_ and -β_2_ was highly variable in both newly diagnosed and recurrent GBM (Figure 3). However, expression levels (in copy number) were clearly increased compared to non-tumoral brain parenchyma samples. Indeed, as shown in the Figure 3A, TGF-β_1_ expression level was increased by 27.8-fold in newly diagnosed and 39.1-fold in recurrent GBM specimens, compared to normal samples. Likewise, compared to normal samples, TGF-β_2_ expression was increased by 3.1- and 20.1-fold in newly diagnosed and recurrent tumors, respectively (Figure 3B).

The median expression of TGF-β_1_ was more than three times greater than TGF-β_2_ in newly diagnosed tumors (Figure 3C). Intriguingly, recurrent tumors had significantly higher levels of both TGF-β isoforms than newly diagnosed GBM.

This suggests that either tumor progression in the course of the disease or treatments offered at first presentation upregulates TGF-β isoforms expression although this could also be explained by the high inter-patient variability. Indeed, TGF-β_1_ expression increased by 1.4-fold whereas TGF-β_2_ mRNA levels strikingly increased by more than 6-fold. However, although TGF-β_2_ was more expressed than TGF-β_1_ in recurrent GBM, the difference was not significant (Figure 3D). Altogether, these results show that the expression of TGF-β_1_ and TGF-β_2_ is significantly greater in glioblastoma than in non-tumoral brain samples. More so, although recurrent GBM express higher levels of both isoforms, it seems that TGF-β_1_ is the prevailing isoform in newly diagnosed tumors (treatment naive samples).

### 2.4. TGF-β Expression and Its Relation to Clinical Surrogates in Newly Diagnosed GBM

**Figure 4:**
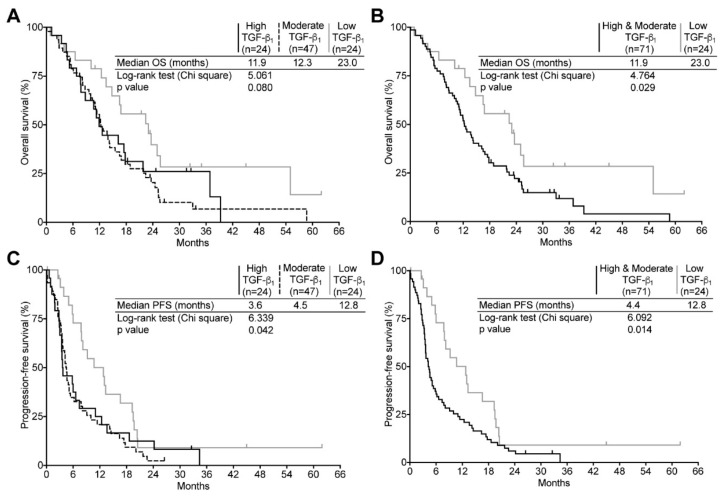

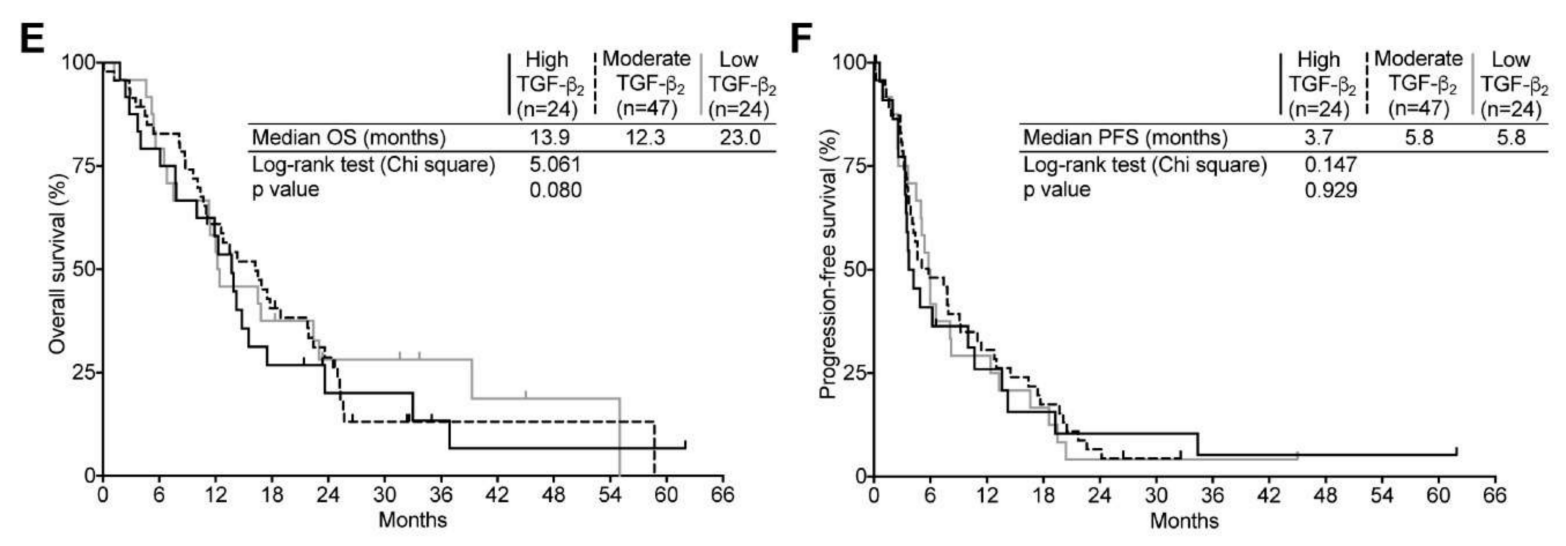
Correlation of TGF-β_1_ and TGF-β_2_ expression level with newly diagnosed GBM patient outcome. Kaplan–Meier estimates of overall survival in newly diagnosed GBM according to three (**A**) high, black line; moderate, dashed line; low, gray line and two (**B**) high + moderate, black line; low, gray line TGF-β_1_ expression subgroups. Kaplan–Meier estimates of progression-free survival in newly diagnosed GBM according to three (**C**) high, black line; moderate, dashed line; low, gray line) and two (**D**) high + moderate, black line; low, gray line subgroups of TGF-β_1_ expression. Kaplan–Meier estimates of overall survival (**E**) and progression-free survival (**F**) according to three subgroups of TGF-β_2_ expression (high, black line; moderate, dashed line; low, gray line).

Based on their relative TGF-β_1_ and -β_2_ mRNA levels, the 95 newly diagnosed tumor specimens were partitioned into three subgroups: high, moderate, and low as described under the TCGA analysis. The expression levels were then correlated with OS and PFS. We found that TGF-β_1_ expressions strongly correlated with a poorer prognosis. Since both the high and moderate TGF-β_1_ expressing subgroups were not statistically different (*p* = 0.57), the two subgroups were merged together in following analyses (Figure 4A,B). We found that the OS of high and moderate TGF-β_1_ expressing tumors was significantly lower than for the low TGF-β_1_ expressing subgroup. Indeed the median OS was 11.9 months for the high/moderate TGF-β_1_ subgroup (95%, CI: 9.0–14.9) compared to 23.0 months for low TGF-β_1_ expressing tumors (95%, CI: 13.9–32.1). Likewise, patients with high and moderate TGF-β_1_ expression had a significantly poorer PFS than low TGF-β_1_ expressing GBM. As depicted in the Figure 4C,D, relapse occurred at a median time of 12.8 months (95%, CI: 7.2–18.3) in low-TGF-β_1_ expressing GBM, whereas in high and moderate TGF-β_1_ expressing tumors, recurrence was observed nearly three times earlier (PFS = 4.4 months (95%, CI: 3.5–5.4)). The multivariate analyses showed results consistent with our Kaplan–Meier survival estimates, indicating that high/moderate TGF-β_1_ expression increased the risk of death and tumor recurrence by 2.005- and 2.167-fold respectively compared to low TGF-β_1_ levels (Tables S2 and S3). Kaplan–Meier and multivariate analyses revealed that TGF-β_2_ expression did not affect either OS or PFS (Figure 4E,F as well as Tables S2 and S3). Our results show that in newly diagnosed GBM, the expression of the predominantly expressed TGF-β_1_, but not TGF-β_2_, is significantly associated to tumor progression and survival and could thus impact the patient’s outcome.

### 2.5. TGF-β Expression and Its Relation to Clinical Surrogates in Recurrent GBM

**Figure 5:**
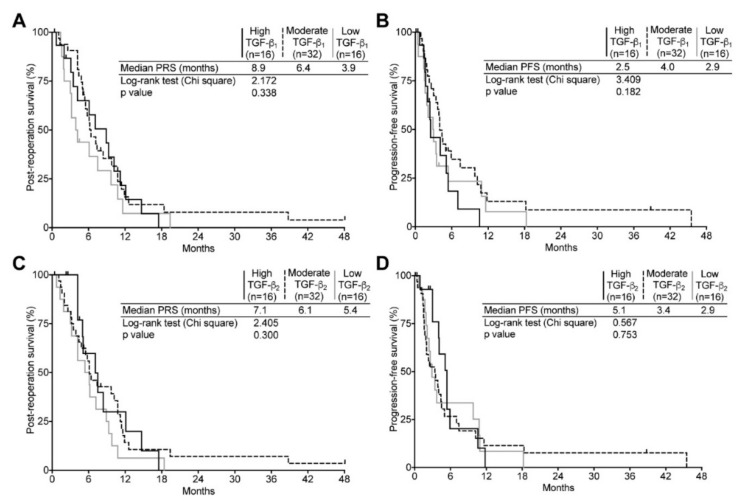
Correlation of TGF-β_1_ and TGF-β_2_ expression level with recurrent GBM patient outcome. Kaplan–Meier estimates of overall survival and progression-free survival in recurrent GBM according to three subgroups (high, black line; moderate, dashed line; low, gray line) of TGF-β_1_ (**A**,**B**) or TGF-β_2_ (**C**,**D**) expression.

The same analysis was repeated for the recurrent tumor group. However, since our cohort comprised seven secondary GBM and our goal was to assess the impact of TGF-β levels on survival of relapsing tumor patients, post-reoperation survival (PRS) was used as surrogate instead of OS. Interestingly, in spite of a substantial increase in both TGF-β_1_ and -β_2_ expression (Figure 3C,D), we uncovered no significant impact on clinical determinants. Indeed, the median PRS and PFS were very comparable for the high, moderate and low TGF-β_1_ and TGF-β_2_ expressing subgroups (Figure 5). Likewise, Cox regressions revealed that although high and moderate TGF-β_1_ expression seemed to confer a somewhat lower risk of death or recurrence, we found these not to be significant (Tables S4 and S5).

Although the number of patients is very low, expression levels for both TGF-β_1_ and TGF-β_2_ were compared in 13 paired samples. Unsurprisingly, there was a lot of variation between patients. Moreover, whereas TGF-β_1_ appeared to be very moderately modulated from the first to the second instance, TGF-β_2_ seemed generally upregulated (Figure S3).

## 3. Discussion

TGF-β appears to be involved somehow in almost all phenotypic attributes of malignant gliomas, thereby highlighting its candidacy as a premium potential target [12]. Unsurprisingly, in this context, TGF-β has received the attention of researchers and clinicians in the field. The relationship between the influence of TGF-β on glial tumor evolution and progression is well acknowledged in the neuro-oncology literature. Traditionally, TGF-β_2_ has been the preferred isoform for its role in glioma genesis and clinical trials specifically targeting this isoform have been carried out [25]. However, this treatment strategy seems to have lost the interest of researchers to the benefit of LY2157299, a TGF-β receptor 1 kinase inhibitor, which has yet to prove its efficiency (ClinicalTrial.gov identifier NCT01220271) [26].

Interestingly, our data suggest otherwise. Indeed, in this work we observed that TGF-β_1_ expression appears to play a more prominent role, but only in newly diagnosed tumors. The expression level of this isoform was three-fold that of TGF-β_2_ and significantly associated with OS and PFS. Moreover, our analysis of the correlation between TGF-β levels and target gene expression also shows a significant increase in the TGF-β pathway activity.

This suggests that TGF-β_1_ mRNA levels could be explored as a prognostic biomarker in newly diagnosed and treatment-naive patients. Evidently, TGF-β_1_ expression could also represent a target of significance in this context. The situation is otherwise in relapsing patients. In this group, the expression levels for TGF-β_1_ mRNA lose their significance in relation to clinical surrogates, whereas TGF-β_2_ expression remains uncorrelated to either PRS or PFS. Noteworthy, however, is the fact that median expression levels of both isoforms increases significantly compared to naive tumors (Figure 3); this increase is especially striking for TGF-β_2_ (six-fold), even if it does not bear significance to clinical surrogates. This phenomenon could result from progression-related genomic remodeling and instability [27]. However, one cannot overlook the possibility that these observations could be treatment-related effect. Indeed, in pre-clinical studies, we have shown that TGF-β_1_ levels were upregulated by radiation therapy in a rodent glial animal model as well as in vitro using human glioblastoma cells [28,29]. In the clinic, radio- and chemotherapy are offered following surgery as part of the standard of care in newly diagnosed tumors. This has a great impact on autocrine regulation of expression and on TGF-β protein and pathway activation since the latent TGF-β_1_, in contrast to latent TGF-β_2_, can be activated by reactive oxygen species generated by ionizing radiation [22,30]. TGF-β upregulation at relapse could derive from a similar effect, being influenced by treatment modalities. These findings also suggest that TGF-β could play an important role in the initial phases of malignant glial tumor progression, a role that is minimized once the tumor has already been exposed to treatment. The amino acids sequences of TGF-β isoforms are highly similar and all three proteins bind to the same receptors to activate intracellular signaling resulting in comparable downstream effects, the differences residing in cell-specific and development-dependent expression [31,32]. Based on the similarity between TGF-β isoforms discussed above, we acknowledge the intrinsic ambiguity involving a specific isoform in playing a dominant role. This observation is hard to explain. However, our analyses were quite clear, and the dichotomic impact of the two TGF-β isoforms on survival was statistically strong. Thus, our results suggest that in designing a targeted therapeutic strategy against TGF-β, one should consider targeting the TGF-β_1_ isoform, and use this approach at primary onset.

Although we did not assess the possibility for TGF-β_3_ to be used as a prognostic biomaker or target, analysis of the TCGA data by Seystahl and collaborators revealed that low expression of this isoform was associated with a better prognosis in GBM of the neural subtype whereas it bore no significant effect in proneural, mesenchymal, or classical GBM subtypes. Moreover, their study corroborated that oligonucleotide-based specific inhibition of TGF-β_3_ significantly reduced invasiveness in vitro as well as in vivo [33].

Our methodology in the current research is rigorous. We have carefully selected samples for which extraction yielded quality RNA (assessed by RIN evaluation) to minimize acquisition of misleading data during mRNA quantification. Moreover, we used a stringent algorithm to normalize qPCR data to compensate for inter- and intra-assay variations. Furthermore, our cohort is very homogenous as all patients were monitored by the same oncology team and we performed multivariate analyses using several clinical variables to guarantee unbiased interpretations of Kaplan–Meier investigations. Our study is, however, burdened with a notable weakness: we have deliberately omitted extending our quantification to the protein level because we were concerned that the recognized tumor heterogeneity inherent to GBM might yield contradictory results and thus compromise the interpretation of our data (i.e., FFPE and qPCR-assessed samples were perhaps not harvested from matching tumor areas). We acknowledge that concurrent mRNA and protein expression analysis could refine our understanding of how TGF-β impacts GBM progression and clinical evolution. However, our analyses of the correlation between TGF-β isoforms and target genes mRNA levels (TCGA data) revealed a significantly increased pathway activity in tumors with high-TGF-β_1_, but not TGF-β_2_ or -β_3_, expression. This supports our observations that TGF-β_1_ is the predominant isoform in the context of GBM clinical evolution.

A recent study by Frei and colleagues focused on analyzing the expression as well as activation of the TGF-β signaling pathway in glioma samples [24]. In a cohort of 64 newly diagnosed patients, they reported that TGF-β_1_ mRNA was significantly more expressed than TGF-β_2_ and -β_3_ which is consistent with our findings. Moreover, they found no significant difference between mRNA levels of all three isoforms in a smaller cohort of 15 recurrent tumors. This is different from our results where TGF-β_2_ expression was significantly higher than in newly diagnosed GBM, even if it did not translate into clinical significance. They also found, both in the newly and the relapsing group, that the expression of all three isoforms correlated with one another. In contrast, at the protein level, TGF-β_2_ was the dominant isoform in both newly diagnosed and recurrent GBM; however, no meaningful correlation was observed between mRNA and protein expression. They also reported significant, although weak, correlations between TGF-β isoforms, pSmad2, pSmad1/5/8, as well as target genes PDGFB or PAI-1 mRNA levels, emphasizing the link between TGF-β and glial oncogenicity.

However, contrary to our work, Frei and coworkers did not find any correlation between any TGF-β isoform expression, either at the mRNA or the protein level, and clinical surrogates. This divergence could be attributed, to a lesser extent, by their lower sample size, but most importantly to the fact that their cohorts were separated in two subgroups (high vs. low TGF-β expression) rather than in our three subdivisions. This suggests that the cutoff values selected for the segregation of the cohort can impact the analyses. To this end, for further assessment of the potential for TGF-β to be a prognostic biomarker, we recommend that a validation cohort and a predetermined cutoff be used. Their analysis of TCGA data, however, revealed that TGF-β gene and target gene expression were higher in GBM with the mesenchymal gene expression signature which is proposed to be associated with reduced OS [24,34].

Although their work did not uncover a predominant TGF-β target, this cytokine remains intricately associated with the malignant phenotype of GBM. It plays several roles in gliomagenesis, most of which occur through the canonical TGF-β signaling pathway. But TGF-β is a very promiscuous cytokine, and when its receptor complex is activated, it can interact and activate other recognized GBM-promoting oncogenes such as PI3K and RAS in non-canonical fashion [35]. Moreover, several migration-inducing genes, such as JNK, p38, and RhoA, which severely impacts cytoskeletal remodeling, expression of extracellular-matrix components, as well as invasion are also targets of the non-canonical TGF-β signal transduction [36]. In addition, pro-angiogenic factors such as Nox4 and TGF-α are downstream targets of non-Smad TGF-β signaling [37].

Finally, several clinical trials have tackled TGF-β inhibition as a treatment strategy both directly, that is at the gene and protein level, as well as indirectly, through the inhibition of its signaling pathway (ClinicalTrials.gov Identifier: NCT00761280, NCT01472731, or NCT02423343). Moreover, results from such trials have yet to prove the benefit of TGF-β inhibition [38]. However, FDA-approved drugs such as chloroquine and pirfenidone have been used to inhibit TGF-β-induced cell growth and epithelial-mesenchymal transition (EMT) and have shown promising results [29,39,40,41]. We believe our study is important because it allows to redefine the parameters of TGF-β research in relation to glial tumors. Indeed, based on our results, we feel that this long-lasting salient query could be solved by focusing on TGF-β_1_ mRNA expression, instead of TGF-β_2_, and by targeting GBM tumors at first presentation, rather than at relapse.

## 4. Materials and Methods

### 4.1. TCGA Data Analysis

Clinical and RNA expression datasets were downloaded via the Data Portal from the glioblastoma multiform dataset of the TCGA network (http://cancergenome.nih.gov/, accessed on 28 February 2018). We used the RNASeqV2 level 3 data (all three batches from UNC IlluminaHiseq_RNASeqV2; RSEM data: RNA-Seq by Expectation Maximization) for expression and Kaplan–Meier analyses [42]. Overall survival, progression-free survival, and other clinical surrogates were found in the clinical and clinical follow-up data files respectively. Data from patients with secondary or recurrent GBM were not analyzed because of the very low number of samples available [43].

### 4.2. Tumor Specimens Acquisition and Diagnosis

Tumor specimens were acquired as described previously [29]. Fresh samples were immediately minced, transferred into RNAlater RNA Stabilization Reagent (QIAGEN, Hilden, Germany) and frozen (−80 °C). All of our samples were acquired between the years 2011 and 2015 (protocol ID #11-088, approved 4 October 2011 by the research ethics committee of the Centre Hospitalier Universitaire de Sherbrooke. Pathological diagnosis was determined by the neuropathologist of our team using the histologic criteria in accordance with the 2007 WHO classification of brain tumors. To name just a few, the IDH1/2, ATRX or *O*^6^-alkylguanine DNA alkyltransferase (MGMT) status is thus unavailable for most of these specimens. Only patients with a diagnosis of GBM were retained for this study.

### 4.3. Non-Tumoral Tissue

Samples from 18 non-tumoral tissue sampled from white matter, kindly provided by the Douglas-Bell Canada Brain Bank (Douglas Mental Health University institute, Montreal, QC, Canada), were also studied for comparison purposes. These sample were collected from patients who died from chronic obstructive pulmonary disease, cardiac or vascular diseases or sudden deaths with no evidence of past medical history.

### 4.4. RNA Extraction and Quality Assessment

Total RNA was extracted from tumor specimens weighing between 40 and 50 mg. Briefly, TRIzol reagent (Invitrogen, Carlsbad, CA, USA) was used as described by the manufacturer up until collecting and transferring the aqueous phase in an RNeasy Mini Spin Column (QIAGEN) to isolate and purify RNA according to the recommended protocol. RNA integrity (RIN) was then assessed using RNA Nano Chips with an Agilent 2100 Bioanalyzer (Agilent Technologies, Santa Clara, CA, USA) by the RNomics platform at our center (available online: http://rnomics.med.usherbrooke.ca/en/). Specimens with RIN lower than 6.5 were not used for further analysis. However, in the case of recurrent GBM and non-tumoral brain samples, we had to lower the RIN threshold to 6.0 and 5.0, respectively, to maximize the number of analyzed samples.

### 4.5. Reverse Transcription and Real-Time Polymerase Chain Reaction (qPCR)

Reverse transcription reactions and qPCR analysis were performed in triplicates as described previously [29]. The sequences of DNA oligonucleotides and probes used for qPCR assays can be found in Table S6. Normalized relative quantity of TGF-β_1_ and -β_2_ were calculated following the mathematical models described by Hellemans and collaborators [44]. HPRT, SFRS9 and TPB were used as reference genes.

### 4.6. Statistics

To assess the correlation of TGF-β expression with OS, PRS and PFS, expression values for TGF-β isoforms of both newly diagnosed and recurrent GBM were stratified in the following subgroups: high, moderate and low. No statistical model was used to define the groups. As we wanted to segregate high-TGF-β from the low-TGF-β expressing tumors, we used the 75th and 25th percentiles as cut-off values (high ≥ 75th > moderate ≥ 25th > low). Partitioning of the data was done prior to analysis and was therefore not data-driven. We used the Log-rank (Mantel-Cox) test for the comparison of Kaplan–Meier curves (Prism 6, GraphPad Software, La Jolla, CA, USA). As this study is exploratory rather than confirmatory, we elected not to use the Bonferroni correction in our analyses containing more than two groups [45]. Comparisons of TGF-β isoforms expression levels were accomplished by one-way analysis of variance (Friedman test) and the Dunn’s multiple comparison test (Prism 6, GraphPad Software, La Jolla, CA, USA). Comparisons of TGF-β isoforms expression levels between GBM subclass and TGF-β target gene expression between TGF-β expression subgroups were accomplished by non-parametric Kruskal Wallis and Mann–Whitney (IBM SPSS Statistics Software, Armonk, NY, USA). Uni- and multivariate analyses, using several clinical surrogates such as age, KPS, tumor location, extent or resection and treatment modalities were completed with the Cox regression model; only the factors identified as significant by univariate analyses were carried over in the multivariate analyses (IBM SPSS Statistics Software).

## Supplementary Materials

Supplementary materials can be found at http://www.mdpi.com/1422-0067/19/4/1113/s1. Figure S1. TGF-β expression in the different GBM subclass of the TCGA dataset; Figure S2. Assessment of the TGF-β pathway activation in newly diagnosed GBM of the TCGA dataset; Figure S3. Comparison of TGF-β expression in paired tumor specimen; Table S1. Patients characteristics; Table S2. Univariate and multivariate analyses for overall survival in newly diagnosed GBMs; Table S3. Univariate and multivariate analyses for progression-free survival in newly diagnosed GBMs; Table S4. Univariate and multivariate analyses for post-reoperation survival in recurrent GBMs; Table S5. Univariate and multivariate analyses for progression-free survival in recurrent GBMs; Table S6. List of genes and sequences of primers & probes used in the qPCR experiments.

## Abbreviations

TGF-β	Transforming growth factor-beta
GBM	Glioblastoma
qPCR	Quantitative real-time polymerase chain reaction
OS	Overall survival
PRS	Post-reoperation survival
PFS	Progression-free survival
