# Supplementary material for: Differential Expression and Clinical Significance of Transforming Growth Factor-Beta Isoforms in GBM Tumors
Source: Int J Mol Sci. 2018 Apr 8;19(4):1113. doi: 10.3390/ijms19041113 (PMC5979513; doi:10.3390/ijms19041113)
Supplement: Supplementary file 1 [file ijms-19-01113-s001.zip › Supplementary Figure S3.pdf]

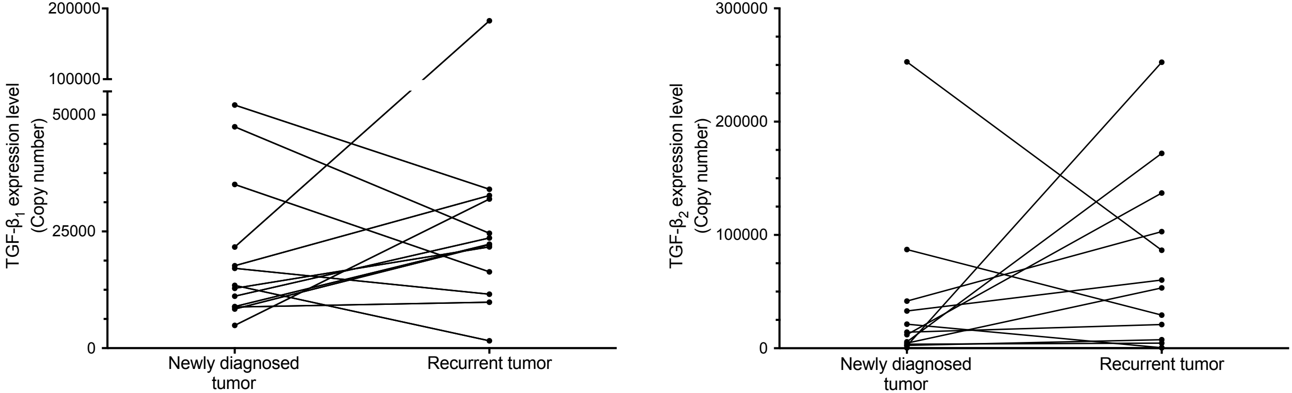

C

	**TGF-β_1**			**TGF-β_2**		
	Level at diagnosis	Level at recurrence	Fold increase/decrease	Level at diagnosis	Level at recurrence	Fold increase/decrease
Patient 1	47436	24616	-0.48	87083	29299	-0.66
Patient 2	12805	21684	0.69	32916	60192	0.83
Patient 3	8914	22265	1.50	2501	7546	2.02
Patient 4	4870	31943	5.56	4540	53274	10.73
Patient 5	17656	32706	0.85	41656	102896	1.47
Patient 6	8821	9853	0.12	14219	20938	0.47
Patient 7	35069	16345	-0.53	11974	137035	10.44
Patient 8	13449	1569	-0.88	367	504	0.37
Patient 9	21667	182583	7.43	21261	427	-0.98
Patient 10	52098	34050	-0.35	3597	4509	0.25
Patient 11	8386	22157	1.64	2142	252427	116.85
Patient 12	17106	11547	-0.32	5651	172012	29.44
Patient 13	11137	23612	1.12	252807	86503	-0.66

Supplementary figure 3. Comparison of TGF-β expression in paired tumor specimen. Comparison of mRNA levels (copy) of TGF-β_1(A) and TGF-β_2(B) as well as fold increase/decrease (C) in tumor sample from subsequent surgeries (n=13).
