# Supplementary material for: Differential Expression and Clinical Significance of Transforming Growth Factor-Beta Isoforms in GBM Tumors
Source: Int J Mol Sci. 2018 Apr 8;19(4):1113. doi: 10.3390/ijms19041113 (PMC5979513; doi:10.3390/ijms19041113)
Supplement: Supplementary file 1 [file ijms-19-01113-s001.zip › Supplementary Figure S1.pdf]

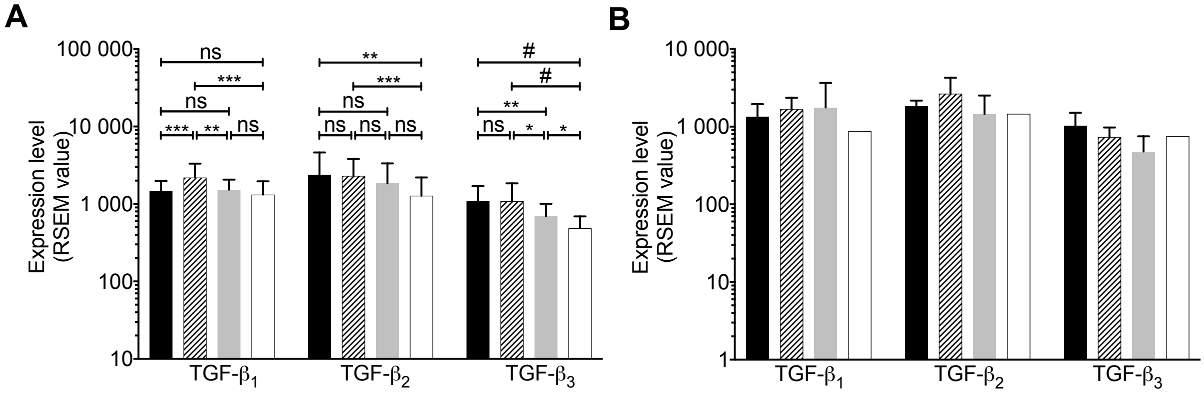

Supplementary figure 1. TGF-β expression in the different GBM subclass of the TCGA dataset. Comparison of mRNA levels (RSEM values) of all three TGF-β isoforms in the classical (black bar), mesenchymal (dashed bar), neural (grey bar) and proneural (white bar) newly diagnosed (A) or recurrent (B) GBM subclass. *, $p < 0.05$; **, $p < 0.01$; ***, $p < 0.001$; #, $p < 0.0001$; ns, not significant.
