# Supplementary material for: Differential Expression and Clinical Significance of Transforming Growth Factor-Beta Isoforms in GBM Tumors
Source: Int J Mol Sci. 2018 Apr 8;19(4):1113. doi: 10.3390/ijms19041113 (PMC5979513; doi:10.3390/ijms19041113)
Supplement: Supplementary file 1 [file ijms-19-01113-s001.zip › Supplementary Table S1.pdf]

Supplementary Table 1. Patients characteristics.

	Newly diagnosed (n=95)	Recurrent (n=64)
**Age at diagnosis (years)**		
Median	62	53
Range	23-83	26-75
**Age classes, n (%)**		
50 years or younger	21 (22.1)	27 (42.2)
51-60 years	24 (25.3)	19 (29.7)
61-70 years	26 (27.4)	14 (21.9)
70 years or older	24 (25.3)	4 (6.3)
**Sex, n (%)**		
Female	44 (46.3)	27 (42.2)
Male	51 (53.7)	37 (57.8)
**KPS (pre-operative), n (%)**		
90-100	20 (21.1)	9 (14.1)
70-80	27 (28.4)	21 (32.8)
50-60	31 (32.6)	14 (21.9)
50 or lower	17 (17.9)	20 (31.3)
**Tumor location at diagnosis, n (%)**		
Frontal	43 (45.3)	22 (34.4)
Temporal	15 (15.8)	13 (20.3)
Parietal	16 (16.8)	6 (9.4)
Occipital	0 (0)	1 (1.6)
Not circumscribed to one lobe	21 (22.1)	22 (34.4)
Right hemisphere	56 (58.9)	27 (42.2)
Left hemisphere	37 (38.9)	37 (57.8)
Not circumscribed to one hemisphere	2 (2.1)	0 (0)
Deep seeded	4 (4.2)	2 (3.1)
**Extent of resection, n (%)**		
Gross total	51 (53.7)	16 (25)
Partial	39 (41.1)	43 (67.2)
Biopsy	5 (5.3)	5 (7.8)
**Histological subtype, n (%)**		
Glioblastoma	69 (72.6)	55 (85.9)
Glioblastoma with oligodendroglial component	22 (23.2)	8 (12.5)
Glioblastoma with PNET component	1 (1.1)	0 (0)
Glioblastoma with gemistocytic component	2 (2.1)	0 (0)
Glioblastoma with small cell component	1 (1.1)	0 (0)
Gliosarcoma	0 (0)	1 (1.6)
**Fisrt line therapy, n (%)**		
Stupp	71 (74.7)	52 (81.3)
RT alone	4 (4.2)	5 (7.8)
RTsc alone	12 (12.6)	2 (3.1)
TMZ alone	2 (2.1)	0 (0)
No treatment	6 (6.3)	2 (3.1)
**Second line therapy, n (%)**		
TTF	1 (1.1)	0 (0)
TMZ alone	34 (35.8)	22 (34.4)
GK	0 (0)	3 (4.7)
IAC	13 (13.7)	33 (51.6)
CCNU	4 (4.2)	2 (3.1)
Avastin	4 (4.2)	0 (0)
No treatment	37 (38.9)	4 (6.3)
N/A	2 (2.1)	0 (0)
Received third line therapy or more	22 (23.2)	26 (34.4)
**Survival (months)**		
Median OS (95% CI; events)	13.6 (11.3-16.8; 78)	24.5 (16.9-26.6; 56)
Median PRS (95% CI; events)	N/A	5.5 (4.4-7.2; 56)
Median PFS (95% CI; events)	5.1 (4.1-7.4; 87)	3.0 (2.3-4.0; 55)
Alive at last followup, n (%)	17 (17.9)	8 (12.5)

PNET, primitive neuroectodermal tumour; RT, radiotherapy; RTsc, short course radiotherapy, TMZ, temozolomide; TTF, tumour treating fields; GK, gamma-knife; IAC, intra-arterial chemotherapy; N/A, not applicable; OS, overall survival; PRS, post-reoperation survival; PFS, progression-free survival.
