# Supplementary material for: Differential Expression and Clinical Significance of Transforming Growth Factor-Beta Isoforms in GBM Tumors
Source: Int J Mol Sci. 2018 Apr 8;19(4):1113. doi: 10.3390/ijms19041113 (PMC5979513; doi:10.3390/ijms19041113)
Supplement: Supplementary file 1 [file ijms-19-01113-s001.zip › Supplementary Table S2.pdf]

Supplementary table 2. Univariate and multivariate analyses for overall survival in newly diagnosed GBMs. Only the variables with a significant p value for the univariate analysis were included in the multivariate analysis. KPS, Karnofsky Performance Status; NRQ, normalized relative quantity; Celldex, Rindopepimut - injectable peptide vaccine targeting epidermal growth factor receptor variant III (EGFRvIII); TTF, tumour treating fields; Metmab, onartuzumab - monoclonal antibody against hepatocyte growth factor receptor (c-Met).

	**Univariate**			**Multivariate**		
**Variables**	**Hazard Ratio**	**C.I. 95%**	**p value**	**Hazard Ratio**	**C.I. 95%**	**p value**
Gender (M vs F)	0.895	0.570 - 1.405	0.630			
Age	1.036	1.017 - 1.055	< 0.001	1.028	1.006 - 1.049	0.011
KPS (preoperative)	0.984	0.970 - 0.998	0.025	0.998	0.981 - 1.015	0.810
**mRNA expression data**						
NRQ TGF-β1	1.017	0.904 - 1.144	0.782			
2 Subgroups TGF-β1 (High + Mod. vs Low)	1.842	1.055 - 3.215	0.032	2.005	1.105 - 3.620	0.022
NRQ TGF-β2	1.015	0.838 - 1.229	0.881			
3 Subgroups TGF-β2 (High vs Low)	1.258	0.670 - 2.362	0.576			
3 Subgroups TGF-β2 (Moderate vs Low)	1.070	0.614 - 1.867	0.810			
**Tumor location**						
Frontal (yes vs no)	0.659	0.418 - 1.038	0.720			
Temporal (yes vs no)	0.754	0.461 - 1.232	0.259			
Parietal (yes vs no)	1.661	1.018 - 2.710	0.042	1.406	0.795 - 2.486	0.242
Occipital (yes vs no)	1.423	0.651 - 3.289	0.410			
Multifocal (yes vs no)	0.465	0.064 - 3.359	0.448			
Right hemisphere (yes vs no)	1.076	0.679 - 1.703	0.756			
Left hemisphere (yes vs no)	0.850	0.534 - 1.353	0.492			
Deep seeded (yes vs no)	2.138	0.777 - 5.886	0.141			
**Extent of resection**						
Gross total (yes vs no)	0.862	0.551 - 1.350	0.518			
Partial (yes vs no)	1.060	0.673 - 1.668	0.803			
Biopsy (yes vs no)	1.588	0.633 - 3.980	0.324			
**Treatment modality**						
Stupp (yes vs no)	0.191	0.109 - 0.334	< 0.001	0.239	0.051 - 1.131	0.071
Celldex (yes vs no)	0.878	0.402 - 1.917	0.745			
TTF (yes vs no)	0.934	0.129 - 6.749	0.946			
Radiotherapy (yes vs no)	3.599	1.272 - 10.180	0.016	0.749	0.130 - 4.314	0.746
Short course radiotherapy (yes vs no)	3.626	1.831 - 7.180	< 0.001	0.634	0.129 - 3.130	0.576
Temozolomide alone (yes vs no)	0.584	0.360 - 0.945	0.029	0.502	0.297 - 0.848	0.010
Gamma knife (yes vs no)	1.314	0.181 - 9.524	0.787			
Intra-arterial chemotherapy (number of cycles)	0.898	0.819 - 0.984	0.021	0.905	0.822 - 0.996	0.041
Metmab (yes vs no)	0.947	0.344 - 2.606	0.916			
CCNU (yes vs no)	0.575	0.286 - 1.158	0.121			
Avastin (yes vs no)	1.222	0.586 - 2.550	0.593			
Combined (yes vs no)	0.988	0.586 - 1.666	0.964			
No treatment (yes vs no)	4.187	1.665 - 10.528	0.002	0.527	0.082 - 3.429	0.504
