# Supplementary material for: Differential Expression and Clinical Significance of Transforming Growth Factor-Beta Isoforms in GBM Tumors
Source: Int J Mol Sci. 2018 Apr 8;19(4):1113. doi: 10.3390/ijms19041113 (PMC5979513; doi:10.3390/ijms19041113)
Supplement: Supplementary file 1 [file ijms-19-01113-s001.zip › Supplementary Table S3.pdf]

Supplementary table 3. Univariate and multivariate analyses for progression-free survival in newly diagnosed GBMs. Only the variables with a significant p value for the univariate analysis were included in the multivariate analysis. KPS, Karnofsky Performance Status; NRQ, normalized relative quantity; Celldex, Rindopepimut - injectable peptide vaccine targeting epidermal growth factor receptor variant III (EGFRvIII); TTF, tumour treating fields; Metmab, onartuzumab - monoclonal antibody against hepatocyte growth factor receptor (c-Met).

	**Univariate**			**Multivariate**		
**Variables**	**Hazard Ratio**	**C.I. 95%**	**p value**	**Hazard Ratio**	**C.I. 95%**	**p value**
Gender (M vs F)	0.759	0.492 - 1.173	0.214			
Age	1.026	1.009 - 1.043	0.003	1.018	0.998 - 1.038	0.073
KPS (preoperative)	0.997	0.984 - 1.011	0.690			
**mRNA expression data**						
NRQ TGF-β1	1.020	0.914 - 1.137	0.726			
2 Subgroups TGF-β1 (High + Mod. vs Low)	1.867	1.128 - 3.091	0.015	2.167	1.277 - 3.676	0.004
NRQ TGF-β2	0.997	0.828 - 1.201	0.979			
3 Subgroups TGF-β2 (High vs Low)	1.027	0.562 - 1.877	0.930			
3 Subgroups TGF-β2 (Moderate vs Low)	0.934	0.562 - 1.551	0.792			
**Tumor location**						
Frontal (yes vs no)	0.604	0.394 - 0.926	0.021	1.166	0.68 - 1.993	0.573
Temporal (yes vs no)	0.822	0.522 - 1.293	0.397			
Parietal (yes vs no)	2.120	1.329 - 3.382	0.002	2.005	1.120 - 3.590	0.019
Occipital (yes vs no)	1.850	0.883 - 3.877	0.103			
Multifocal (yes vs no)	1.508	0.365 - 6.230	0.570			
Right hemisphere (yes vs no)	0.924	0.600 - 1.424	0.721			
Left hemisphere (yes vs no)	0.850	0.534 - 1.353	0.857			
Deep seeded (yes vs no)	3.722	1.327 - 10.443	0.013	5.652	1.95 - 16.415	0.001
**Extent of resection**						
Gross total (yes vs no)	1.033	0.674 - 1.582	0.883			
Partial (yes vs no)	0.891	0.576 - 1.377	0.603			
Biopsy (yes vs no)	1.550	0.625 - 3.846	0.344			
**Treatment modality**						
Stupp (yes vs no)	0.325	0.189 - 0.558	< 0.001	0.554	0.256 - 1.199	0.134
Celldex (yes vs no)	0.819	0.409 - 1.642	0.574			
TTF (yes vs no)	1.149	0.159 - 8.316	0.891			
Radiotherapy (yes vs no)	1.192	0.597 - 6.117	0.275			
Short course radiotherapy (yes vs no)	2.973	1.528 - 5.784	0.001	1.59	0.635 - 3.981	0.322
Temozolomide alone (yes vs no)	0.785	0.511 - 1.205	0.268			
Gamma knife (yes vs no)	0.869	0.120 - 6.281	0.890			
Intra-arterial chemotherapy (number of cycles)	0.971	0.905 - 1.042	0.411			
Metmab (yes vs no)	0.797	0.291 - 2.183	0.659			
CCNU (yes vs no)	0.679	0.375 - 1.231	0.121			
Avastin (yes vs no)	1.468	0.705 - 3.056	0.305			
Combined (yes vs no)	0.983	0.599 - 1.613	0.944			
No treatment (yes vs no)	1.950	0.707 - 5.377	0.197			
