# Supplementary material for: Differential Expression and Clinical Significance of Transforming Growth Factor-Beta Isoforms in GBM Tumors
Source: Int J Mol Sci. 2018 Apr 8;19(4):1113. doi: 10.3390/ijms19041113 (PMC5979513; doi:10.3390/ijms19041113)
Supplement: Supplementary file 1 [file ijms-19-01113-s001.zip › Supplementary Table S4.pdf]

Supplementary table 4. Univariate and multivariate analyses for post-reoperation survival in recurrent GBMs. Only the variables with a significant p value for the univariate analysis were included in the multivariate analysis. KPS, Karnofsky Performance Status; NRQ, normalized relative quantity; Metmab, onartuzumab - monoclonal antibody against hepatocyte growth factor receptor (c-Met).

	**Univariate**			**Multivariate**		
**Variables**	**Hazard Ratio**	**C.I. 95%**	**p value**	**Hazard Ratio**	**C.I. 95%**	**p value**
Gender (M vs F)	0.965	0.561 - 1.661	0.899			
Age	1.017	0.996 - 1.039	0.117			
KPS (preoperative)	0.983	0.968 - 0.999	0.041	0.984	0.967 - 1.001	0.057
**mRNA expression data**						
NRQ TGF-β1	0.961	0.800 - 1.153	0.665			
3 Subgroups TGF-β1 (High vs Low)	0.717	0.343 - 1.497	0.376			
3 Subgroups TGF-β1 (Moderate vs Low)	0.623	0.329 - 1.179	0.246			
NRQ TGF-β2	0.824	0.648 - 1.048	0.114			
3 Subgroups TGF-β2 (High vs Low)	0.617	0.285 - 1.340	0.222			
3 Subgroups TGF-β2 (Moderate vs Low)	0.637	0.341 - 1.188	0.156			
**Tumor location**						
Frontal (yes vs no)	0.898	0.525 - 1.536	0.694			
Temporal (yes vs no)	1.066	0.620 - 1.834	0.816			
Parietal (yes vs no)	1.751	0.972 - 3.156	0.062			
Occipital (yes vs no)	1.217	0.515 - 2.878	0.655			
Right hemisphere (yes vs no)	0.965	0.553 - 1.682	0.899			
Left hemisphere (yes vs no)	1.061	0.613 - 1.836	0.833			
Deep seeded (yes vs no)	3.246	0.763 - 13.802	0.111			
**Extent of resection**						
Gross total (yes vs no)	0.553	0.282 - 1.085	0.085			
Partial (yes vs no)	1.349	0.746 - 2.439	0.322			
Biopsy (yes vs no)	2.047	0.800 - 5.233	0.135			
**Treatment modality**						
Stupp (yes vs no)	0.652	0.256 - 1.661	0.370			
Radiotherapy (yes vs no)	2.148	0.512 - 9.002	0.296			
Temozolomide alone (yes vs no)	0.568	0.285 - 1.133	0.109			
Intra-arterial chemotherapy (number of cycles)	0.856	0.778 - 0.941	0.001	0.855	0.780 - 0.937	0.001
Metmab (yes vs no)	0.935	0.128 - 6.838	0.948			
CCNU (yes vs no)	0.532	0.226 - 1.249	0.147			
Avastin (yes vs no)	0.534	0.240 - 1.188	0.124			
Combined (yes vs no)	0.558	0.262 - 1.188	0.130			
No treatment (yes vs no)	6.288	1.827 - 21.637	0.005	2.779	0.764 - 10.103	0.121
