# Supplementary material for: Differential Expression and Clinical Significance of Transforming Growth Factor-Beta Isoforms in GBM Tumors
Source: Int J Mol Sci. 2018 Apr 8;19(4):1113. doi: 10.3390/ijms19041113 (PMC5979513; doi:10.3390/ijms19041113)
Supplement: Supplementary file 1 [file ijms-19-01113-s001.zip › Supplementary Table S5.pdf]

Supplementary table 5. Univariate and multivariate analyses for progression-free survival in recurrent GBMs. Only the variables with a significant p value for the univariate analysis were included in the multivariate analysis. KPS, Karnofsky Performance Status; NRQ, normalized relative quantity; Metmab, onartuzumab - monoclonal antibody against hepatocyte growth factor receptor (c-Met).

	**Univariate**			**Multivariate**		
**Variables**	**Hazard Ratio**	**C.I. 95%**	**p value**	**Hazard Ratio**	**C.I. 95%**	**p value**
Gender (M vs F)	1.931	1.100 - 3.390	0.022			
Age	1.002	0.983 - 1.021	0.827			
KPS (preoperative)	0.967	0.953 - 1.007	0.231			
**mRNA expression data**						
NRQ TGF-β1	1.136	0.940 - 1.373	0.187			
3 Subgroups TGF-β1 (High vs Low)	1.127	0.520 - 2.446	0.762			
3 Subgroups TGF-β1 (Moderate vs Low)	0.635	0.334 - 1.208	0.166			
NRQ TGF-β2	0.943	0.759 - 1.173	0.599			
3 Subgroups TGF-β2 (High vs Low)	0.824	0.373 - 2.833	0.633			
3 Subgroups TGF-β2 (Moderate vs Low)	1.081	0.575 - 2.031	0.809			
**Tumor location**						
Frontal (yes vs no)	1.158	0.673 - 1.992	0.597			
Temporal (yes vs no)	0.914	0.528 - 1.583	0.749			
Parietal (yes vs no)	2.199	1.135 - 4.259	0.020	1.695	0.829 - 3.466	0.148
Occipital (yes vs no)	0.645	0.253 - 1.644	0.358			
Right hemisphere (yes vs no)	1.139	0.655 - 1.982	0.645			
Left hemisphere (yes vs no)	0.856	0.494 - 1.484	0.580			
Deep seeded (yes vs no)	0.226	0.030 - 1.710	0.150			
**Extent of resection**						
Gross total (yes vs no)	0.496	0.251 - 0.981	0.044	0.549	0.252 - 1.196	0.131
Partial (yes vs no)	1.437	0.780 - 2.649	0.245			
Biopsy (yes vs no)	4.415	1.429 - 13.644	0.010	2.043	0.625 - 6.672	0.237
**Treatment modality**						
Stupp (yes vs no)	1.002	0.394 - 2.548	0.997			
Radiotherapy (yes vs no)	4.094	0.946 - 17.720	0.059			
Temozolomide alone (yes vs no)	0.451	0.230 - 0.885	0.021	0.389	0.189 - 0.799	0.010
Intra-arterial chemotherapy (number of cycles)	0.950	0.869 - 1.039	0.265			
Metmab (yes vs no)	2.455	0.329 - 18.307	0.381			
CCNU (yes vs no)	0.669	0.301 - 1.488	0.324			
Avastin (yes vs no)	1.053	0.494 - 2.245	0.894			
Combined (yes vs no)	1.139	0.553 - 2.346	0.724			
No treatment (yes vs no)	2.468	0.577 - 10.557	0.223			
