# Supplementary material for: Differential Expression and Clinical Significance of Transforming Growth Factor-Beta Isoforms in GBM Tumors
Source: Int J Mol Sci. 2018 Apr 8;19(4):1113. doi: 10.3390/ijms19041113 (PMC5979513; doi:10.3390/ijms19041113)
Supplement: Supplementary file 1 [file ijms-19-01113-s001.zip › Supplementary Table S6.pdf]

Supplementary table 6. List of genes and sequences of primers & probes used in the qPCR experiments.

Gene	Forward	Reverse	Probe
Transforming Growth Factor-beta 1 (TGF-β_1) - NM_000660	5′-GCCTTTCCTGCTTCTCATGG-3′	5′-TCCTTGCGGAAGTCAATGTAC-3′	5′-/56-FAM/CCGAGCCCTG GACACCAACTAT/3IABkFQ/-3′
Transforming Growth Factor-beta 2 (TGF-β_2) - NM_001135599	5′-GTGCCTGAACAACGGATT-3′	5′-ATTCGCCTTCTGCTCTTG-3′	5′-/5MAXN/AACATCTCCAAC CCAGCGCTACAT/3IABkFQ/-3′
Hypoxanthine Phosphoribosyltransferase (HPRT) - NM_000194.2	5′-GACTTTGCTTTCCTTGGTCAG-3′	5′-GGCTTATATCCAACACTTCGTG-3′	5′-/56-FAM/ATGGTCAAGGTCGC AAGCTTGCTGGT/3IABkFQ/-3′
Serine and Arginine Rich Splicing Factor 9 (SFRS9) - NM_003769	5′-TGTGCAGAAGGATGGAGT-3′	5′-CTGGTGCTTCTCTCAGGATA-3′	5′-/5MAXN/TGGAATATGCCCT GCGTAAACTGGA/3IABkFQ/-3′
TATA-box Binding Protein (TBP) - NM_003194.4	5′-CACGAACCACGGCACTGATT -3′	5′-TTTTCTTGCTGCCAGTCTGGAC-3′	5′-/56-FAM/TGTGCACAGGAGC CAAGAGTGAAGA/3IABkFQ/-3′
