# Supplementary material for: Differential Expression and Clinical Significance of Transforming Growth Factor-Beta Isoforms in GBM Tumors
Source: Int J Mol Sci. 2018 Apr 8;19(4):1113. doi: 10.3390/ijms19041113 (PMC5979513; doi:10.3390/ijms19041113)
Supplement: Supplementary file 1 [file ijms-19-01113-s001.zip › Supplementary Figure S2.pdf]

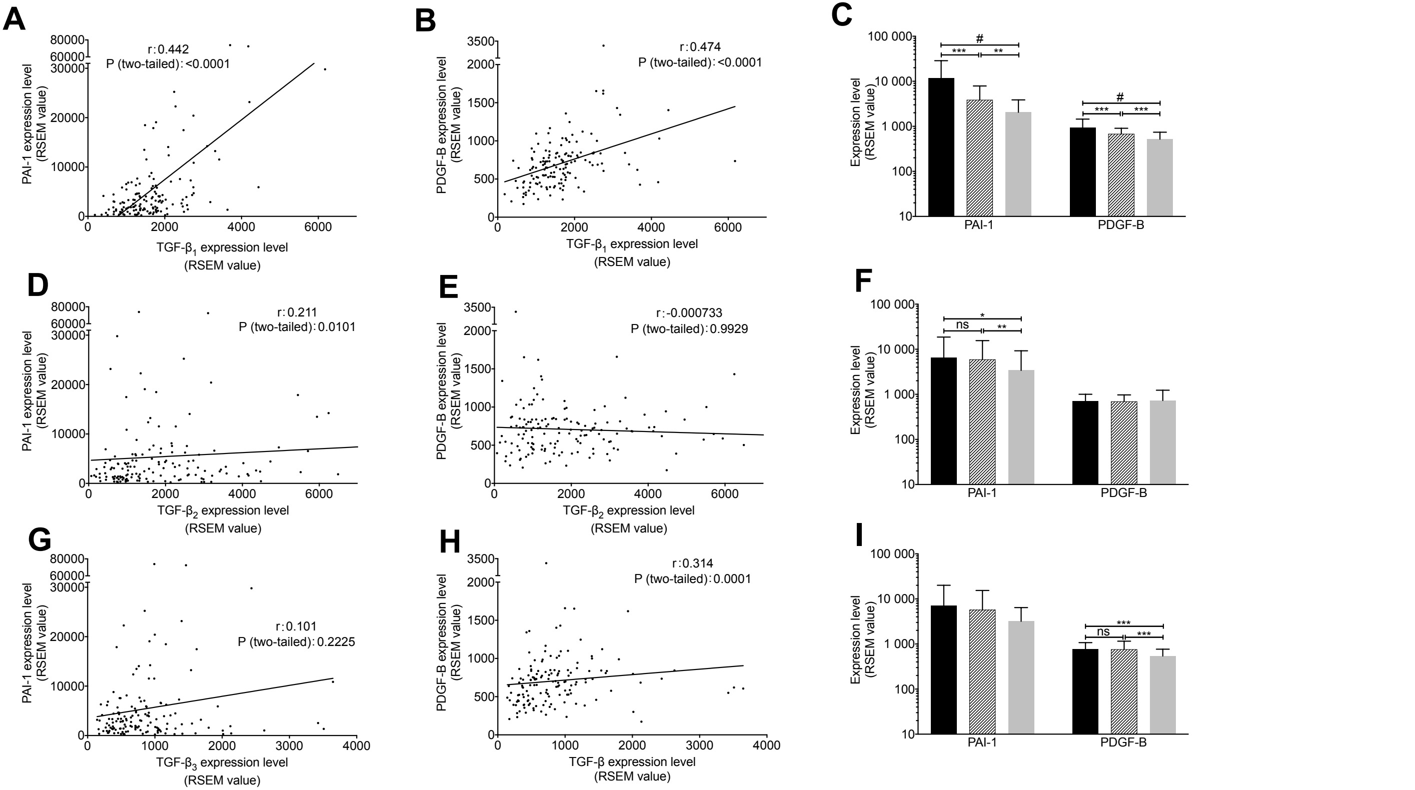

Supplementary figure 2. Assessment of the TGF-β pathway activation in newly diagnosed GBM of the TCGA dataset. Correlation of TGF-β_1, TGF-β_2 or TGF-β_3 mRNA levels with the expression of TGF-β target genes PAI-1 (A, D, G) and PDGF-B (B, E, H). Comparison of TGF-β target genes mRNA levels (RSEM values) in three subgroups (high, black bar; moderate, dashed bar; low, white bar) of TGF-β_1,TGF-β_2 or TGF-β_3 expression (C, F, I).
